# Effectiveness of congenital myelodysplastic clubfoot treatment by the Ponseti method—Systematic review

**DOI:** 10.1371/journal.pone.0304909

**Published:** 2024-10-04

**Authors:** Tatiana Ferreira dos Santos, Gabriel Ferraz Ferreira, Monica Paschoal Nogueira

**Affiliations:** 1 Foot and ankle surgeon, Postgraduate in Instituto de Assistência Médica ao Servidor Público Estadual (IAMSPE) São Paulo, São Paulo, Brazil; 2 Instituto Vita, São Paulo, São Paulo, Brazil; 3 Pediatric Orthopaedics and Reconstruction Group, Department of Orthopaedics and Traumatology, Instituto de Assistência Médica ao Servidor Público Estadual (IAMSPE) São Paulo, São Paulo, Brazil; Policlinico Universitario A. Gemelli IRCCS - Universita Cattolica del Sacro Cuore Roma, ITALY

## Abstract

In myelomeningocele children, the incidence of equinocavovarus feet, considering all foot deformities, is 25–36%. Treatment options consist of extensive surgeries resulting in rigid feet with better alignment. Ponseti method expanded its indications since the early 2000s, including myelodysplastic feet. However, the literature on success, recurrence, and complication rates remains sparse. Therefore, a systematic review was performed in Pubmed, Scopus, Embase, Lilacs, and Web of Science databases on October 28, 2020 and July 11, 2023. Normality and sample proportion analysis with 95% confidence intervals were estimated. Risks of bias and the quality of studies were also evaluated. Success, recurrence, and complication rates were evaluated and analyzed. Eight case series were identified with 101 patients (176 feet). According to this model, the initial success rate was 93% (95% CI = 0.88–0.96) with I^2^ = 0%, and the final success was 63% (at 4.9 years of follow-up). Recurrence rate was 62% (95% CI = 50–72), and complication rate was 29% (95% CI = 22–38). Ponseti method for myelodysplastic clubfoot is effective (93% of initial correction). However, there are high complication and recurrence rates, and longer follow-up is needed to identify recurrences and urge for early intervention. Foot abduction brace should be used to avoid recurrences.

## Introduction

Meningomyelocele is a congenital malformation in the spinal cord, present in 1.9 for every 10,000 live births. It is due to neural tube defects in the first weeks of pregnancy characterized by a cystic formation containing nerve roots [1–4]. This defect represents 86,8% of all neural tube defects.

Congenital talipes equinovarus (clubfoot) is a musculoskeletal deformity that accounts for 40% (30%–50%) of all foot deformities in meningomyelocele children [5–9]. This deformity in these patients results from failure of neuromuscular control for neurological lesions (in idiopathic clubfoot, it results from muscular contracture and defect of soft tissue composition). Surgical treatment is the accepted and indicated method to treat this condition, with extensive releases of soft tissues and bone resections. According to the literature, this treatment has high morbidity and good results, between 63% and 76% of all techniques. Serial casts were contraindicated for these patients before the 2000s, mainly because they were insensitive and stiffer feet. The Ponseti method was initially proposed due to its high success rates [1, 10–12], which are similar to conventional treatment but have less morbidity. This study is a systematic review of the Ponseti method applied to treat congenital myelodysplastic clubfoot, whose objective is to show the effectiveness and low morbidity of the method and its success, complication, and recurrence rates.

## Methods

### Search strategy

A systematic review was performed by two reviewers (TFS and GFF) according to the Preferred Reporting Items for Systematic Reviews and Meta-analysis (PRISMA) guidelines [13].

The search was conducted on Pubmed, Lilacs, Scopus, Web of Science, and Embase databases. The search was performed on 28 October 2020, and a new search was conducted on 11 January 2023 using the keywords "myelomeningocele" and "clubfoot" with a boolean search AND without any language or date restriction or any filter. The first strategy was a broad search because the topic is recent, and we knew there might be few articles. Additionally, an active search was performed in the cited articles in the revised papers to ensure all articles were included. The gray literature was searched in Google Scholar without results. The reviewers retrieved the data and independently analyzed each selected study. The PRISMA figure shows the selection of these papers.

Disagreements were resolved by the senior investigator (MPN).

### Inclusion and exclusion criteria

The inclusion criteria were: (1) studies including clinical diagnosis of myelomeningocele and clubfoot; (2) studies describing treatment by Ponseti Method; (3) studies describing minor surgical procedure associated with the surgical Achilles tenotomy or anterior tibial transfer; (4) studies with clubfoot without other previous surgical treatment.

The exclusion criteria were: (1) studies describing other neuromuscular diseases or syndromes; (2) case reports.

### Data extraction

Two reviewers independently extracted the data according to the following predefined criteria: name of the first author, publication year, country, study design, type of study, number of patients, number of feet, age, and follow-up.

### Quality assessment

The methodological index for non-randomized studies (MINORS) was used to evaluate the methodological quality of the selected studies because all the studies included in this review were observational [14].

Studies with a score ≤ 11 were rated low quality, and studies with scores ≥ 12 were rated high quality.

### Risk and publication bias

A funnel plot was performed to evaluate the publication bias.

### Statistical analysis

A meta-analysis of proportions was performed with data normalized using the logit function based on the selected outcomes. Heterogeneity was calculated using the i^2^. The random effects model was selected for the meta-analysis, and the calculations were performed using the R software.

### Data items

The results were synthesized and categorized using the Mendeley tool, and duplicates were removed.

### Outcomes

The outcomes selected for evaluation were success, recurrence, and complication rates. The success rate was evaluated twice: in the final of the serial casts and (or not) Achilles tenotomy and in the last follow-up. Success was defined as a plantigrade foot, with no deformities, braceble, with no need for additional procedures.

Three of the eight studies showed mixed results with the other primary pathologies. The data was obtained from meningomyelocele patient’s data and discussions presented in the articles. Because of that, some data were not evaluated.

This systematic review was registered on PROSPERO under the number CRD42021244300.

## Results

### Eligible studies

The search retrieved 734 articles: Pubmed (91), Embase (212), Scopus (384), Web of Science (44), and Lilacs (03). Duplicated articles were excluded, and after each article was carefully analyzed (letters, conference proceedings, and book chapters were first excluded). Then, all papers were analyzed considering the inclusion and non-inclusion criteria.

**Fig 1:**
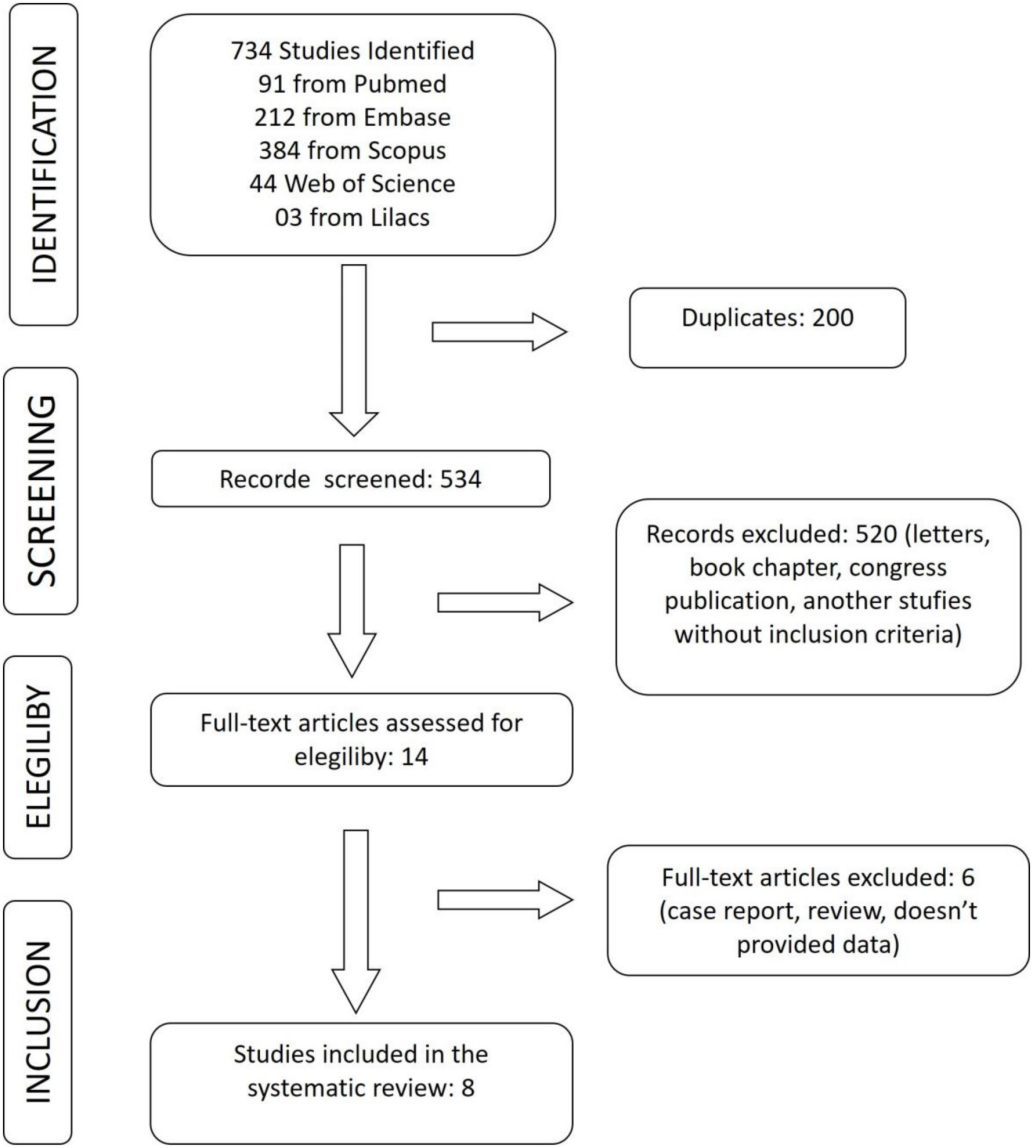
PRISMA flowchart of the literature search and study selection.

After the analysis, eight observational studies that met the established criteria were selected for the meta-analysis. The flowchart representing the study selection is shown in Fig 1.

### Demographic characteristics of the included studies

The studies were published from 2009 to 2018. Eight case series were identified: two from the United States [15, 16], two from the United Kingdom [7, 17], one from Canada [18], one from Egypt [12], one from Italy [19], and one from India [20]. There were 101 patients (176 feet) aged 1 to 24 weeks old. One study had one child at 103 weeks old. Follow-up was from one to nine years (Table 1).

**Table 1:** Demographic and treatment characteristics of the included studies.

Study	Year	Country	Study design	Number of patients	Feet	Both feet	Initial treatment age (weeks)	Follow-up (Years)
Gerlach et al. [15]	2009	USA	Prospective case series	16	28	12	12 (1103)	2.8
Janicki et al. [18]	2009	Canada	Retrospective case series	5	9	4	9.2 (4–24)	2.6 (1–3.5)
Dunkley et al. [17]	2015	UK	Prospective case series	15	24	9	11.2	4.6 (2–8)
Matar et al. [7]	2017	UK	Retrospective case series	11	18	9	4.7 (2–8)	4.5 (3–9)
El-Fadl et al. [12]	2016	Egito	Prospective case series	24	48	24	5.9 (3–8)	2.25 (2–2.8)
Arkin et al. [16]	2018	USA	Retrospective case series	17	26	7	6.5 (3–18)	5.4 (1.8–7.8)
Abraham et al. [19]	2021	Italy	Retrospective case series	8	15	7	23	3.26
Sharma et al. [20]	2021	India	Prospective case series	5	8	3	9.8 (1–21)	9
TOTAL	.	.	.	101	176	75	10.2*	4.3*

*Mean

Ponseti method was chosen as the treatment method for all patients. In most cases, casts were applied by the senior researcher, but one study had casts applied by physiotherapists. Moreover, the type of brace used after the cast phase differs; this difference is shown in Tables 1 and 2.

**Table 2:** Classification and treatment characteristics of the included studies.

Study	N of patients	N of feet	Classification		N of casts	Achilles tendon tenotomy (feet)	Mitchell Boot and Barr*	Regular FAB	Dynamic brace **
			Pirani	Demeglio					
Gerlach et al. [15]^1^	16	28	N/S	3.3 (2–4)	5 (4–8)	85.7%		8	7
Janicki et al. [18]	5	9	N/S	N/S	4.2 (3–6)	77.7%		5	
Dunkley et al. [17]	15	24	5.5 (3–6)	N/S	7	N/S	15		
Matar et al. [7]	11	18	5.5 (3.5–6)	N/S	7 (4–9)	94%	11		
El-Fadl et al. [12]^2^	24	48	N/S	3.41 (3–4)	6.8	100%		24	
Arkin et al. [16]^3^	17	26	N/S	N/S	6.54 (2–9)	88.4%		17***	
Abraham et al. [19]	8	15	N/S		4,7	100%		8	
Sharma et al. [20]	5	8	5 (4–6)		8,22	N/S		5	
TOTAL	101	176	5.5	3.4	6.1	91%			

FAB: Foot abduction brace, N/S: Not specified.

*original Ponseti brace,

**brace allowing more foot mobility,

***regular FAB changed to AFOs due to skin problems.

^1^one patient with congenital femoral deficiency and not able to use FAB,

^2^the author performed Z-plasty instead tenotomy,

^3^the author changed the protocol, in 11 patients was performed Achilles tendon tenectomy

Complications were defined as any skin or bone injury in the cast or brace phase. Treatment failure was defined as the need for extensive surgical release with or without associated bone procedures. Recurrence was defined as the appearance of any deformity or dorsiflexion < 0 degrees, based on the information provided in the articles.

Cases included in the studies were evaluated based on clinical assessment. Success was defined as a plantigrade foot without residual deformity, with some dorsiflexion, and no need for additional surgical procedures. Information about correction classification was not provided in all papers, and some information about Pirani classification and others Dimeglio. For that reason, they are not included in success analysis.

The quality of the selected studies was evaluated using MINORS [14], and the summarized study quality assessment is shown in Table 3.

**Table 3:** Summary of the quality of the selected studies using the methodological index for non-randomized studies (MINORS)*.

	Gerlach et al. [15]	Janicki et al. [18]	Dunkley et al. [17]	Matar et al. [7]	El-Fadl et al. [12]	Arkin et al. [16]	Abraham et al. [19]	Sharma et al. [20]
A stated aim	2	2	2	2	2	2	2	2
Inclusion of consecutive patients	2	2	2	2	2	2	2	2
Prospective collection of data	2	1	2	1	2	1	0	2
Endpoints appropriate to the aim of the study	2	2	0	2	2	0	0	1
Unbiased assessment of the study endpoint	0	0	0	0	0	0	0	0
Follow-up period appropriate to the aim of the study	1	1	2	2	1	1	2	2
Loss of follow-up less than 5%	2	2	2	2	0	2	2	2
Prospective calculation of the study size	0	0	0	0	0	0	0	0
Total score**	11	10	10	11	9	8	8	11
Quality of the study***	low	low	low	low	low	low	low	low

*without additional criteria in the case of comparative studies,

**record as 0 (non-reported), 1 (reported but inadequate), or 2 (reported and adequate),

***studies with a total score ≥ 12 were rated as having a high methodological quality

### Pooled analysis

The outcomes success, recurrence, and complication rates show results that made a pooled analysis possible.

### Risk and publication bias

**Fig 2:**
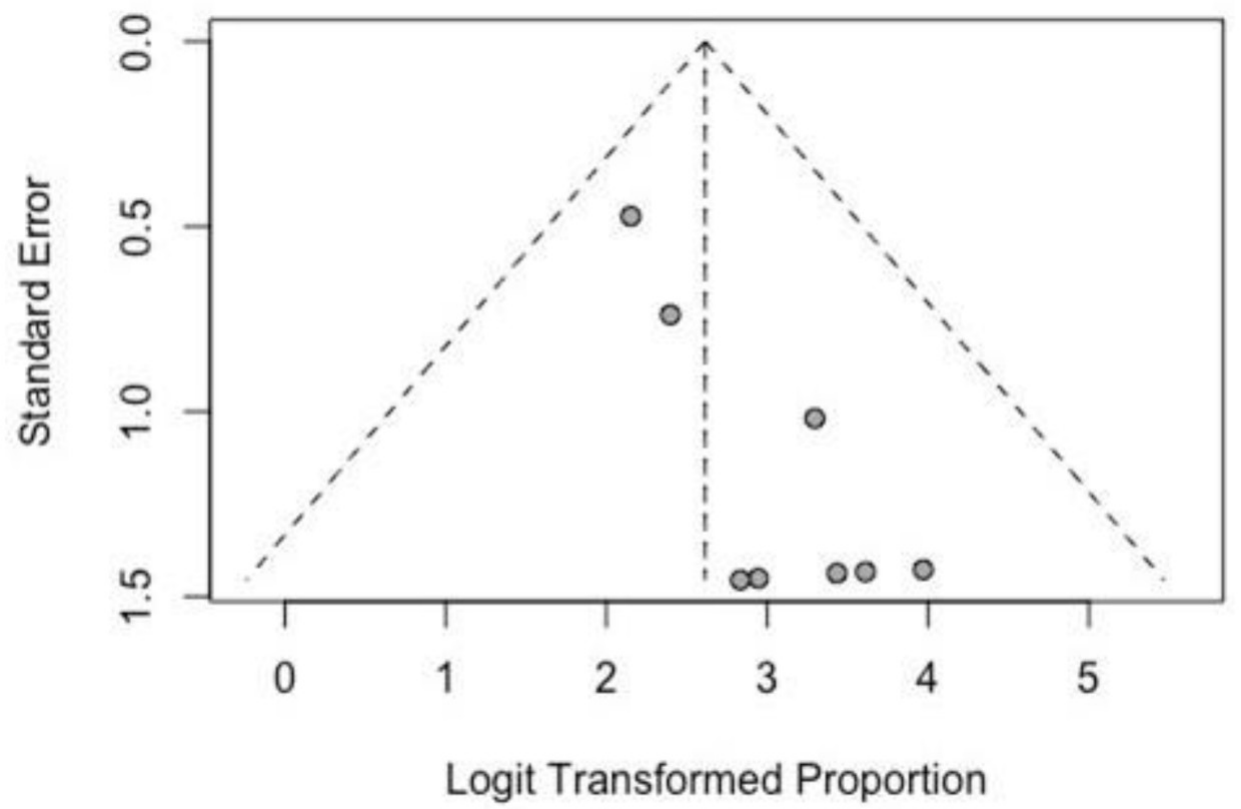
Funnel plot of the publication bias.

Publication bias was minimized for a large search in the database without language restriction and additional search in the gray literature; however, it is not possible to eliminate this bias. Despite a funnel plot being a statistical tool recommended for ten or more studies (according to Cochrane), a funnel plot was performed because the eight included studies are our best evidence. Fig 2 shows qualitative analysis with heterogeneity distribution.

### Outcomes

All studies included were carefully read, and the value of the outcomes is shown in Table 4.

**Table 4:** Outcome results.

Study	N of feet	Initial correction	Final clinical outcome	Complications (feet)	Recurrences
Gerlach et al. [15]	28	27 (96%)	24 (86%)	9 (32.1%)	19 (67.8%)
Janicki et al. [18]	9	9 (100%)	6 (66%)	2 (22%)	5 (55.5%)
Dunkley et al. [17]	24	22 (91.6%)	N/S	N/S	13 (54.1%)
Matar et al. [7]	18	18 (100%)	15 (83.3%)	N/S	8 (44.4%)
El-Fadl et al. [12]	48	43 (89.5%)	N/S	10* (41.6%)	N/S
Arkin et al. [16]	26	26 (100%)	12 (46.1%)	10 (38.4%)	15 (57.7%)
Abraham et al. [19]	15	15 (100%)	N/S	N/S	13 (86.6%)
Sharma et al. [20]	8	8 (100%)	1 (12.5%)	3 (37.5%)	7 (87.5%)
TOTAL	176	168 (93%)	58 (63%)	44 (29%)	80 (62%)

All data refers to the number of feet. N/S: not specified.

*data on the number of patients

### Success

**Fig 3:**
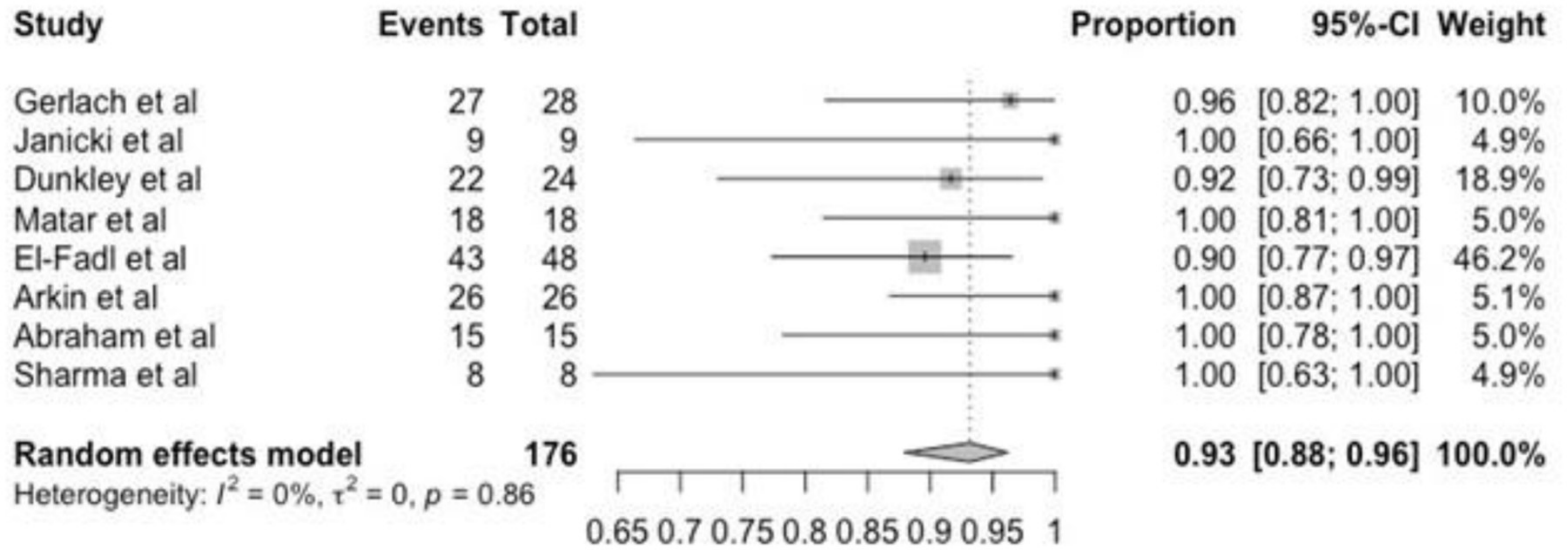
Forest plot of initial success (initial correction).

**Fig 4:**
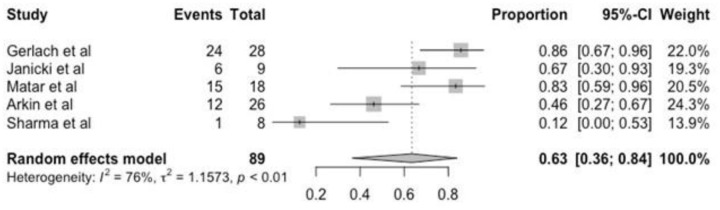
Forest plot of the final success (final clinical outcome).

Success rate was analyzed twice during treatment: after the final cast (initial correction) and at the last follow-up (final clinical outcome). Forest plots summarized the results for initial correction among the eight included studies. Results were compared in all but three studies that mixed other primary pathologies, and data could not be evaluated. Linear and random effect models are similar because there is no heterogeneity (i^2^ = 0%) and show an initial success rate (initial correction) of 93% (95% CI = 0.88–0.96) and the final success rate (final clinical outcome) was 63% with high CI (confidence interval) and high heterogeneity (Figs 3 and 4).

### Recurrence

**Fig 5:**
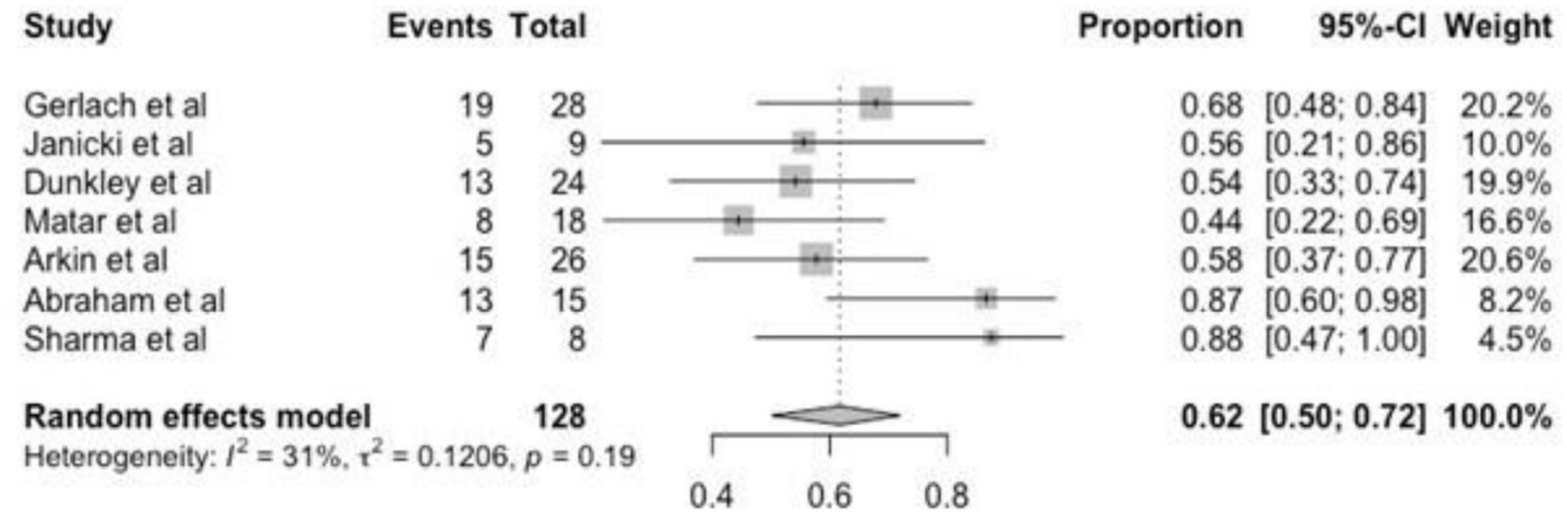
Forest plot of recurrence.

Seven studies were evaluated regarding the recurrence rate, and it was found 62% (95% CI 50–72, p = 0.19) and heterogeneity i^2^ = 31%. One study did not include this information (Fig 5).

### Cast phase complications

**Fig 6:**
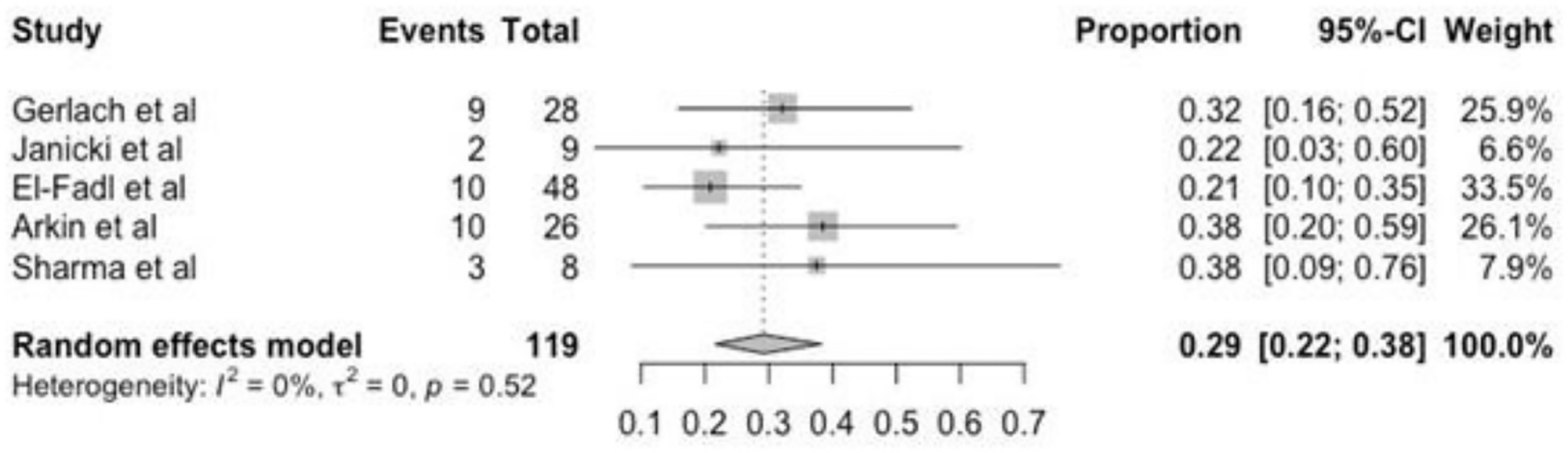
Forest plot of the meta-analysis of studies examining the complications.

Complications associated with the Ponseti method occur in the cast or brace phase. The data for this outcome was dichotomously categorized as present or absent and described as swelling, erythema, fracture, or another injury, as shown in Table 5. Three studies did not include this information. According to the meta-analysis, the complication rate was 29% (95% CI 22–38) (Fig 6).

**Table 5:** Summary of complications.

Study	N of patients	N of feet	Complications (feet)	Type of complication
Gerlack et al. [15]	16	28	9	Fractures, skin lesions, cast slip
Janicki et al. [18]	5	9	2	Skin lesions
Dunkley et al. [17]	15	24	N/S	
Matar et al. [7]	11	18	N/S	
El-Fadl et al. [12]	24	48	10*	Hyperemia and swelling
Arkin et al. [16]	17	26	10	Irritation of skin, skin lesions
Abraham et al. [19]	8	15	N/S	
Sharma et al. [20]	5	8	3	Skin lesions and infection

N/S: Not specified.

* Number of patients

## Discussion

The Ponseti method, the gold standard treatment for treating idiopathic congenital clubfoot, is today an option for treating children with neural tube defects due to its low cost, high initial correction rate, and similar good long-term results compared with surgical treatment [7, 15].

Compared with the idiopathic clubfoot Ponseti method in myelomeningocele treatment has a slightly lower initial correction rate but different long-term results due to the high recurrence rate.

For these patients, the treatment of foot deformity aims to obtain a plantigrade foot with some flexibility and be able to receive an orthosis. Svartman et al. [21] and Shingade et al. [22] showed 71%–76% satisfactory results in patients with arthrogryposis and myelodysplastic clubfoot performing talectomy. Other authors demonstrated similar results using different bone procedures [23–25].

Bone procedures are reserved for severe rigid feet or persistent deformities; then, extensive releases of soft tissues remain the treatment of choice for these feet [26–28], hence the importance of this review, which shows satisfactory results in 63% of cases at the end of follow-up.

This result shows the method’s efficiency in correcting the deformity and the difficulty in maintaining the correction. Unlike the results for idiopathic feet, the recurrence rate is higher in these patients.

The analysis of the funnel plot of the articles shows that more studies are needed.

The systematic analysis demonstrated in forest plots show that treatment with the Ponseti method in these patients is effective because the results are homogeneous in most of the results. Unlike systematic analysis of case-control studies, the funnel plot does not have a null effect line, and a shift to the right in the plot represents greater effectiveness, complication, or recurrence.

In all studies included in this review, the authors discuss foot stiffness and other intrinsic characteristics of these patients, such as muscle imbalance, lack of sensitivity, and proprioception, contributing to these recurrence rates and lower success rates at the end follow-up.

Treatment of these patients should begin with a long and honest conversation with the family, explaining the importance of proper follow-up and the use of orthoses since adherence is an important factor for the success and reduction of recurrences [7, 17].

Lack of adequate use of abduction brace was a factor considered by most authors to explain the recurrence rate, but its direct effect is not clear in all studies. One of them mentions active measures to ensure adequate use of the orthosis with weekly telephone contact performed by a specialized nurse—no other mention of measures specifically aimed at this other than routine guidance during follow-up appointments.

Therefore, as in idiopathic patients, variations of the use of abduction brace may be associated with more recurrences. We consider it an important factor despite insufficient data entailing it.

Regarding the method’s applicability, the studies varied regarding the Achilles tendon tenotomy and the use of orthoses. Achilles tendon tenotomy was performed under local or general anesthesia, according to the surgeon’s preference. El-Fadl et al. [12] performed a Z-plasty for stretching the Achilles tendon, and Arkin et al. [16] performed an Achilles tendon tenectomy after frequent recurrences. On the other hand, the type of abduction orthoses varied according to the author’s preference, and the use of continuous ankle-foot orthoses from 14 hours onwards was a consensus. Despite the variations, the results obtained were similar in getting the initial correction of the feet.

Another important evidence is the type of recurrences. New casts and return of the abduction orthoses are indicated to treat recurrences in idiopathic patients and, therefore, should be for non-idiopathic patients. However, in some studies, the abandonment of conservative treatment and the option for surgical treatment reduced the method’s success rate at the end of follow-up [16, 18, 19].

Still, on the method and considering the intrinsic characteristics already described above, casting in children with meningomyelocele is crucial, and pressure ulcers, hematomas, and edemas are very common. Warning signs such as irritability and pain are usually absent, requiring additional care to avoid skin lesions. Insensitive feet with neglected or rigid deformities are more prone to skin lesions and potentially serious infections. In these cases, the casts act in correcting and protecting, promoting tissue repair and wound healing.

The complication rate was 29%, mainly due to the lack of control of the microvasculature and insensitivity of these feet, leading to increased edema and the potential for skin lesions in sequential casts. Inadequate molding is also responsible for the cast sliding, contributing to complex clubfoot and more skin problems. Fractures were also mentioned and reflect bone fragility.

Regarding the follow-up time of these patients, the authors of this review believe that the follow-up until skeletal maturity in these patients can be an important factor for the reduction of recurrences and improvement in the final success rate. As all the evidence available and included in this review did not have a long enough follow-up time, we believe that new follow-up studies of these patients can demonstrate whether skeletal maturity contributes significantly to reducing recurrences.

Despite the extensive review, this study has limitations. In addition to follow-up time, the studies included in this review are cases of series with low methodological quality, half of them retrospective, which increases the risk of information bias, and recording bias since the data were obtained without a study proposal. Further studies with longer follow-up time are needed to better assess recurrence rates and final correction.

In conclusion, the Ponseti method in patients with myelodysplastic clubfoot presented high rate of initial correction satisfactory results (93%), an excellent option to treat these feet. Still, it is necessary to be aware of the complications and keep in mind that patients have insensitive feet, which are more susceptible to skin lesions and fractures, and require long follow-up time for early diagnosis and intervention in recurrences, in addition to the prolonged use of orthoses.

## Supporting information

S1 Checklist (DOCX)

S1 Table Studies. (XLSX)

S2 Table Sharma data. (XLSX)

S3 Table Article selection. (XLSX)

S4 Table Success. (XLSX)

S5 Table Complications. (XLSX)

S6 Table Recurrence. (XLSX)
